# Evaluation of the Stability of Dielectric Nanofluids for Use in Transformers under Real Operating Conditions

**DOI:** 10.3390/nano9020143

**Published:** 2019-01-23

**Authors:** Victor A. Primo, Daniel Pérez-Rosa, Belén García, Juan Carlos Cabanelas

**Affiliations:** 1 Electrical Engineering Department, Universidad Carlos III de Madrid, 28911, Leganés, Madrid, Spain; vprimo@ing.uc3m.es (V.A.P.); daperezr@ing.uc3m.es (D.P.-R.); 2 Material Science and Engineering Department, Universidad Carlos III de Madrid, 28911, Leganés, Madrid, Spain; caba@ing.uc3m.es

**Keywords:** dielectric nanofluid, long-term stability, Fe_3_O_4_, electrical insulation, power transformer

## Abstract

The application of nanotechnology to the electrical insulation of transformers has become a topic of interest in the last few years. Most authors propose the use of dielectric nanofluids, which are obtained by dispersing low concentrations of nanoparticles in conventional insulating liquids. Although a good number of works have demonstrated that dielectric nanofluids may exhibit superior dielectric properties than the base fluids, there is a key issue that still needs to be addressed, which is the long-term stability of those liquids. The studies about the stability of dielectric nanofluids fluids that have been published so far analyze the performance of the fluids under laboratory conditions which are far from the real working conditions the liquids would be subjected to when working inside a transformer. In this paper, an experimental study is presented that evaluates the stability of several dielectric nanofluids under realistic transformer operating conditions. As the study demonstrates, the stability of dielectric nanofluids depends strongly on the working temperature, on the materials applied to obtain the fluid, and on the manufacturing procedure, while other aspects, such as the interaction with other materials, are less relevant. Additional topics, such as the methods applied for evaluation of the stability and the physical properties of the dielectric nanofluids under test, are discussed in the paper as well.

## 1. Introduction

Power transformers are one of the most expensive and critical elements in electric power systems. Their function is to raise the voltage level of the electrical energy injected to the electrical grid, making it possible to reduce the energy losses associated with the energy transport, and to reduce the voltage level of the energy supplied to the consumers. The reliability of transformers is basic for an adequate operation of electric power systems. Unexpected failures in transformers can lead to supply interruptions and cause great damages to electrical power stations. Some of the main causes of transformer failure are those related to their electrical insulation.

Transformer insulation is responsible for isolating the elements of the transformer with different voltage levels (windings, tank, etc.). The electrical insulation of large and medium-sized power transformers is generally composed of oil-impregnated cellulose (Kraft paper and pressboard pieces) and an insulating fluid, commonly mineral oil (MO). In recent years, the application of alternative fluids such as natural or synthetic esters is becoming common as well, especially in medium-sized units.

Recently, attempts have been made to apply nanotechnology to transformer insulating systems. The application of these systems is aimed at developing transformers capable of handling higher voltage levels, and to transfer greater amounts of power in a safer way, while keeping optimum sizes and costs for the machines. Although a few authors have proposed the development of nanotechnology-based alternatives to traditional solid insulation [1,2], most researchers are working on the development of improved dielectric fluids in which nanoparticles (NP) are incorporated in MO or ester-based liquids.

Dielectric nanofluids (DNF) are two-phase systems composed of a solid-dispersed NP phase and a dielectric liquid used as dispersion medium, generally named base fluid (BF) [3,4]. Different types of NP and BF have been used in previous works. Regarding the NP, three main groups of NPs have been used: conductive NP, such as Fe_3_O_4_; semi-conductive NP, such as TiO_2_, Fe_2_O_3_, ZnO, CuO or CuO_2_, and insulating NP, such as Al_2_O_3_, SiO_2_, or SiO. Typical NP diameters are in the range 10-60 nm [5,6,7,8]. Most publications on DNF are concerned with MO-based fluids, but some works in which natural or synthetic ester-based oils were investigated have been published [9,10,11,12,13,14,15,16].

A good number of works have reported results that prove that the dielectric properties of insulating fluids can be significantly improved by adding low concentrations of NP to them [5,10,17,18,19,20,21]. Different properties, such as the breakdown voltage or the partial discharge inception voltage can be enhanced thanks to the addition of NP [3]. Several authors have suggested that when a dielectric nanofluid (DNF) is subjected to an electric field, positive ionic charges accumulate on the surface of the NP, attracting negative ionic charges around them [22,23]. Thus, an electrical double layer (EDL) is formed. The volume of the EDL close to the NP surfaces is called the compact layer, and consists of immobile negative ions strongly attracted to the NP surfaces. The net charge density in the compact layer drops gradually with increasing distance from the NP surfaces, reaching zero in the electrically neutral area of the fluid. The ions in this region, called the diffuse layer of the EDL, are less affected by the electrostatic interaction with the NP and thus have higher mobility [24]. The formation of the EDL in a DNF reduces the mobility of the electric charges in the DNF, slowing down the breakdown processes in the fluid when it is subjected to electric stress [24,25].

Some authors claim that the application of DNF to power transformers could lead to the obtaining of machines with a more endure insulating system and better prepared to withstand dielectric events such as the over-voltages produced during lighting or switching events [26,27]. However, DNF are not a feasible technical solution nowadays. Issues related to their stability, the interaction with the magnetic fields present in the transformer, the effect of the NP on the transformer solid insulation performance, and the production costs should be studied and addressed before they can be applied in real transformers.

Some of the most referenced problems in the field of nanofluids are those related with their stability [10,21,27,28]. The dispersion of the NP in the BF can be truncated with the passage of time, as NP agglomerate to form particles of micrometric or even macro-metric size [22,29]. The loss of stability of nanofluids is a major problem, since the presence of those aggregates can worsen the properties of nanofluids, giving rise to fluids of poorer properties than those of the BF.

A stable nanofluid is one in which the NP remain homogeneously dispersed in the BF in the long term. In that case, the attractive Van der Waals forces are compensated with other repulsive forces, mainly of electrostatic, steric, and electro-steric nature [30]. The Derjaguin–Landau–Verwey–Overbeek (DLVO) theory is generally used to describe some of the phenomena involved in the stability of nanofluids [31]; according to it a total “interaction potential” can be calculated as the sum of all the attractive and repulsive forces acting between particles. The repulsive forces introduce a barrier in the potential that the particles must surpass to agglomerate. If that energy barrier is bigger than the kinetic energy of the particles, the solution remains stable [30].

A few authors have evaluated the stability of DNF for use in transformers reporting dispersal times varying from hours to several months [12,26,27,32,33]. Most of the works agree in the fact that a low weight fraction of NP implies greater stability than higher weight fractions [34], because as the NP concentration rises, the probability of collision between NP increases and in consequence it is more likely that aggregates are formed. It must be highlighted that all those authors have used visual inspection as evaluation method to determine the stability of the dispersions. As will be latter discussed in this work this evaluation method could not be effective in all cases.

To improve the stability of DNF it is a common practice to use surfactants, which are products that reduce the surface tension in the fluid, influencing the contact area created between two phases. The surfactant covers the NP, preventing the formation of bonds with other NP through steric interactions. Although most authors agree in the suitability of using surfactants as stabilizing agents [35], some works report that the addition of large amounts of surfactant can reduce the dispersal time and worsen the dielectric properties of the DNF [36,37]. In that case, the excess of surfactant would form a double chain around the surface of the NP [38] that could coil up with the surfactant agent that covers the NP forming micelles. With this physics mechanism, the balance between the surfactant effect and the attractive force causes the agglomeration and prevents the NP achieving their objective.

One of the key factors that should be taken into account when analyzing the stability of DNF for use in transformers, is to consider the conditions these materials will be subjected to during their service life. Transformer’s insulating fluids are generally subjected to variable temperature profiles in a typical range 30–110 °C; additionally, they coexist with other materials such as the solid insulation that covers the windings, or the metallic elements that constitute the transformer active part. Although it seems likely that these aspects might have an impact on the stability of DNF (i.e., Ghadimi [31] points out that at temperatures above 60 °C the bonding between surfactant and NP can be damaged what can jeopardize the stability of DNF [17]) it has not been a common practice within the published works, to evaluate the stability of DNF at temperatures different from the ambient one, or to consider the influence of the presence of other materials on the stability.

In this article, an experimental study is presented that analyzes the stability of several Fe_3_O_4_-based DNF manufactured with MO and with a natural ester (NE) as BF. Unlike previous published works this study evaluates the long-term stability of the DNF under real transformer working conditions. The impact of the operating temperature and the interaction with other materials on the stability of the fluids is analyzed. The influence of other aspects, such as the preparation method and the used materials, is discussed as well.

## 2. Materials and Methods

The experimental study presented in this work includes the evaluation of the long-term stability of four groups of DNF. The materials and procedures used to prepare the fluids, and the methodology applied to evaluate their stability are described in this section.

### 2.1. Materials

#### 2.1.1. Base Fluids

Two different commercial transformer oils have been used as BFs: a MO and a NE. The MO was Nytro 4000X, manufactured by Nynas AB (Nynas AB, Stockholm, Sweden), which is composed of 70–90% hydrotreated light naphthenic acid, and 10–30% lubricating oils (C20-50, hydrotreated neutral oil-based) and less than 0.4% 2, 6-di-tert-butyl-p-cresol. This product is commercialized for use in oil-filled electrical equipment, including power and distribution transformers, rectifiers, circuit breakers, and switchgears. The NE was Bioelectra, manufactured by Repsol (Madrid, Spain), which is based on vegetable oils in a percentage higher than 99% and without the presence of synthetic antioxidants. The properties of both insulating liquids are shown in Table 1.

#### 2.1.2. Nanoparticles

Two commercial dispersions of Fe_3_O_4_ NP were used for manufacturing the DNFs under study:

- A dispersion of Fe_3_O_4_ NP of approximate diameter 10 nm dispersed in a hydrocarbon to a concentration of 50% by weight (i.e., 500 g of NP in 1 kg of dispersion) manufactured by the company Magnacol Ltd (Newtown, UK). From now on this dispersion will be referred to as FF1.
- A dispersion of Fe_3_O_4_ NP of diameter 10 nm dispersed in a mixture of hydrocarbons, silicon compounds and non-flammable oils manufactured by the company MAGRON (Gyeonggi-Do, Korea), and commercialized by the company Supermagnete (Gottmadingen, Germany) under the name MFR-DP1. The concentration of this fluid is 60% by weight. From now on this dispersion will be referred to as FF2.

Both dispersions were prepared by the industrial manufacturers using the so-called one-step method in which the NP are simultaneously synthesized and dispersed within the BF [41]. The application of the one-step manufacturing method leads to stable fluids with high concentrations of NP (within 50% and 60% by weight). The NP on those fluids are coated with surfactants to inhibit aggregation. Additionally, working with NP dispersions poses less risk for the workers than using nanopowders [36,42]. One disadvantage of using NP dispersions is that manufacturers sometimes do not provide enough information about their formulation.

**Figure 1:**
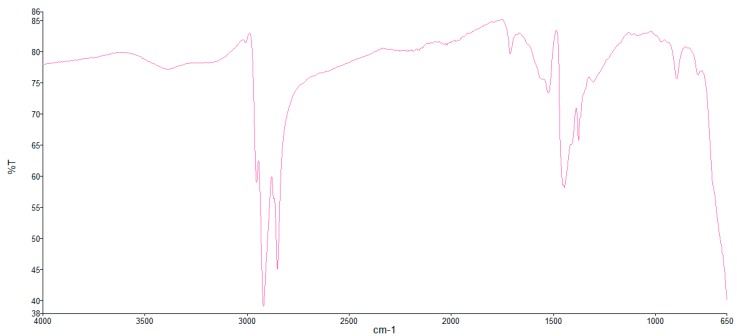
FTIR spectra of FF1.

**Figure 2:**
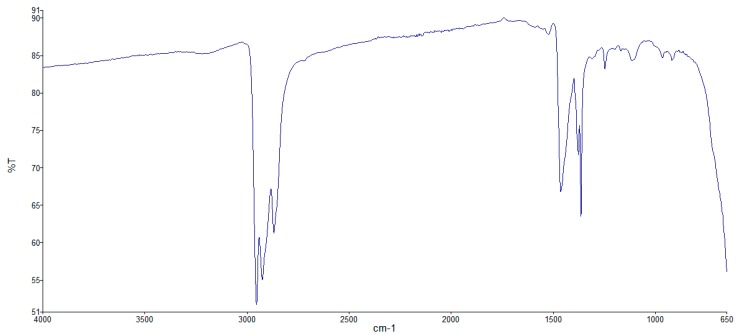
FTIR spectra of FF2.

Samples of FF1 and FF2 were subjected to Fourier-Transform Infrared (FTIR) spectroscopy tests to characterize their composition; the FTIR spectra of both dispersions are shown in Figure 1 and Figure 2.

From these spectra it can be deduced that the carrier liquid is a hydrocarbon in both cases. This is indicated by the sharp peaks around the wavelength 3000 cm^−1^, belonging to C–H stretching spectral vibrations. In the case of FF1, we can affirm that there is a mixture of hydrocarbons, in which the peak at 1700 cm^−1^ usually refers to a carbonyl of a carboxyl group, an amide, or ester. Also, FF1 has a broad band around 3400 cm^−1^ which is compatible with the presence of a carboxyl group or a mixture of a carboxyl group with hydrocarbons, so we can affirm that the carrier liquid of FF1 is a hydrocarbon and that the surfactant is carboxylic acid. In the case of FF2, the carrier liquid is a hydrocarbon as well and the surfactant could not be clearly identified.

Inductive Coupling Plasma (ICP) spectroscopy tests were applied to determine the concentration of Fe and other elements in the NP dispersions obtaining that the concentrations of Fe in FF1 and FF2 were 60 and 50% respectively.

Table 2 summarizes the properties of the two applied dispersions deduced from the described tests and from the information provided by the manufacturers.

### 2.2. Synthesis of the DNF

The preparation of a DNF involves the formation of stable colloids, so that the NP remain suspended in the BF [31,43]. The DNFs analyzed in this work were obtained by adding small amounts of FF1 and FF2 to the BFs up to the desired concentrations, and then subjecting these mixtures to agitation until achieving a homogeneous and stable dispersion.

**Figure 3:**
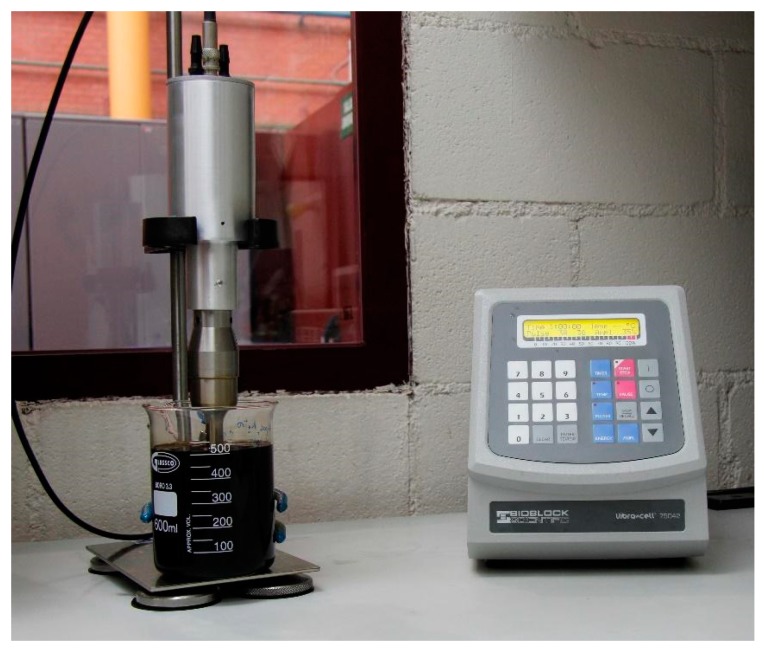
Sonicator 750 W used to produce the DNF.

According to previous works, the method applied to disperse the nanoparticles in the BFs plays a very important role in the obtained results, since it seems to affect the DNF final properties and the long-term stability of the dispersion. Petzold et al. [34] compared the stability of the liquids obtained by using several mixing procedures (ultrasound bath, probe-type ultrasonic devices and magnetic stirrers) finding that the probe-type ultrasonic devices achieved the smaller particle diameters with the lower agitation times. Some other references [30,34], also recommend the use of ultrasound proves instead of ultrasound baths or magnetic stirring. In this work an ultrasonic processor of rated power 750 W and ultrasound wave intensity 670 W/cm^2^, manufactured by the company Sonics & Materials.inc (Newtown, CT, USA) was applied to disperse the NP into the BF (Figure 3).

The sample preparation process applied in this work consists on the following steps:

- The BF (MO or NE) is dried under vacuum (0.1 bar) for 24 h at 70 °C.
- In this work DNF with different concentrations of NP were prepared (although not all of them were tested in the stability study, as will be later explained). To obtain the different concentrations, the required mass of NP dispersion is weighed in an analytical balance and added to the BF. In the case of the MO-based DNF with FF2 the concentrations were 0.05, 0.1, 0.2 and 0.35 g/L (i.e., 0.05 g, 0.1 g, 0.2g and 0.35 g of Fe_3_O_4_ NP in 1 L of BF). To obtain these concentrations 10, 20, 40, and 70 mg of FF2 were added to 100 mL of MO. In the case of NE-based DNF and FF1, concentrations 0.1 and 0.15 g/L were prepared, as samples with higher concentrations precipitated in a few hours. For the combinations MO + FF1 and NE + FF2 only samples with concentration 0.1 g/L were prepared because of the difficulty to obtain stable samples with higher concentrations.
- The mixture is homogenized with the sonicator, at 40% of the rated power (i.e., power 300 W and ultrasound wave intensity 268 W/cm^2^), for two hours in intervals of 30 seconds of agitation and 30 s of pause to avoid overheating the oil.
- The obtained DNF are stored at 50 °C under vacuum (0.1 bar) to avoid oxidation and to remove the moisture absorbed during the mixing process. All the samples are kept in identical conditions until the beginning of the tests.

**Figure 4:**
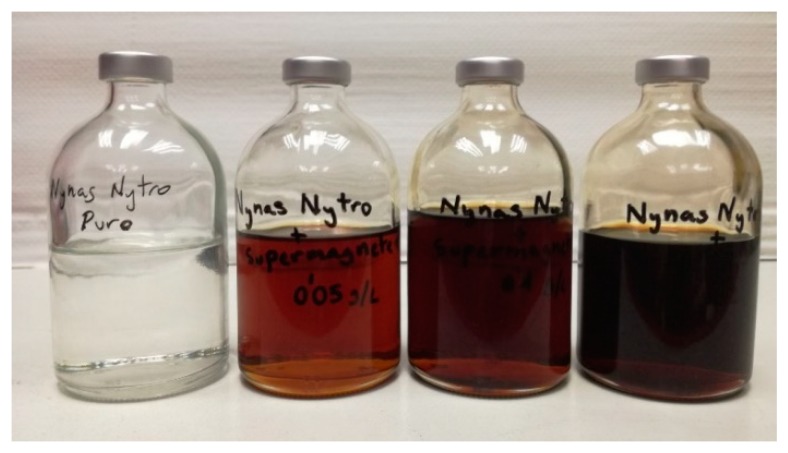
Samples of MO-based DNF with different concentrations of NP. From left to right: MO without NP, MO + FF2 (0.05 g/L), MO + FF2 (0.1 g/L) and MO + FF2 (0.2 g/L).

Figure 4 shows a photography of some of the obtained MO-based DNF.

### 2.3. Experimental Methodology

Unlike previous studies, this work evaluates the stability of the prepared DNF under real operating conditions, i.e., considering the temperatures the fluids would be subjected to when operating inside a service-transformer and the effect of the interaction with other materials that coexist with the fluids inside the transformer.

The operation of a transformer involves a continuous generation of heat derived from the flow of current through the conductors that constitute the transformer windings. One of the missions of the insulating fluid is to dissipate these losses to the ambient. The service temperature of the elements of a transformer depends on the load connected to the transformer, on the ambient temperature, and on the transformer design. The international standard IEEE C57-91-211 [44] analyzes the typical distribution of temperatures inside a transformer as a function of its loading conditions and stablishes that transformer internal temperatures are typically within the range 40 to 110 °C. These temperatures are far from the ambient temperature that has been considered in the stability studies of DNF published to date.

In this work five testing temperatures were considered: 25 °C, 50 °C, 60 °C, 80 °C and 110 °C. To test the stability of the prepared DNF at those temperatures, samples of them were stored in glass vials of 10 mL and kept under controlled temperature conditions for 2 months. In the case of the sample kept at ambient temperature the evaluation time was extended to 10 months. All the possible combinations of NP dispersions and BF were included in the study; then the stability of four DNF was evaluated: MO + FF1, MO + FF2, NE + FF1 and NE + FF2 all of them with NP’s concentration 0.1 g/L. DNF with higher NP concentrations were not included in the study because preliminary tests showed us that the stability of these liquids at temperatures above 25 °C was seriously compromised, and also because the samples with concentration 0.1 g/L demonstrated better global physic-chemical properties that the liquids with higher NP concentrations, as is be explained in Section 3.1.

Another objective of the study was to analyze whether the interaction of the DNF with other materials present inside of the transformer tank had an impact on its stability. The insulating fluid in a transformer coexists with cellulosic materials (i.e., Kraft paper, pressboard, and wood) which act as electrical insulation and support, and with metallic parts (i.e., cooper constituting the windings and iron that constitutes the magnetic core). The present study has analyzed the influence of the cellulosic insulation on the stability of DNF; these materials are in deep contact with the DNF which impregnates them.

Aiming at evaluating this factor, additional testing vials were prepared which included a strip of Kraft paper of mass ≈ 0.5 g. Considering all the previous, eight samples were included for each testing temperature: two samples for each combination of BF and NP dispersion one with Kraft paper in the vial and the other one without paper.

The stability of the samples was evaluated by means of visual inspection and with the measure of the particle size. Visual inspection has been the most commonly applied technique for the evaluation of the stability of DNF for use in transformers [30]. This method is based on the fact that the loss of stability of the fluids is sometimes noticeable by the apparition of aggregates or precipitates in the fluid. On the other hand, the particle or aggregate size can be used as a macroscopic indicator of the interactions between particles. In this way, the size of the aggregate decreases the extent to which the chemical barrier increases, and the aggregation process of the particles becomes more difficult.

Particle-size measurements were performed by dynamic light scattering, using a Malvern Panalytical (Malvern, UK) Zetaziser Nano ZS instrument. This equipment uses non-invasive back scattering. The Brownian movement of the nanoparticles in suspension induces fluctuations of the scattered intensity which are analyzed by a time-auto-correlation function allowing the obtention of a relaxation time related to the diffusion coefficient D of the nanoparticles. Finally, the hydrodynamic radius is calculated using the Stokes-Einstein equation, $R=k_{b}T/6\pi \eta D$, where η is the solvent viscosity and k_b_ is the Boltzmann constant. The samples were illuminated with a He-Ne laser (632.8 nm) and the scattered light was analyzed at a backscattering angle of 173°. For each sample, at least three measurements were done. The software of the equipment analyzes the autocorrelation function to obtain the intensity and, using the Mie theory, the volume size distributions. The temperature at the measuring chamber was adjusted to 25 °C. Equilibration time was 2 min and at least three measurements were done for each sample.

Attempts were done to apply Zeta potential to the evaluation of the stability of the prepared DNF. The measure of the Zeta potential, which is the electric potential on the surface of the NPs and is proportional to the amount of electrical charge attached to the NP surfaces, has been widely used to evaluate the stability of water-based nanofluids [30]. In the case of the oil-based samples studied in this work, Zeta potential measures were not conclusive, since the applied BF are based mainly on hydrocarbons, which are not sufficiently polar, and thus the nanoparticles did not attach enough electrical charge to their surfaces to create a measurable potential.

The possibility of applying the light transmission measurement to the evaluation of the stability was also rated. This technique, based on the fact that NP aggregates have a refractive index greater than that of the medium, was not finally applied because the materials used to prepare the DNF under study absorb in the UV-V what complicates the measurements.

## 3. Results

### 3.1. Physical and Chemical Evaluation of the DNF

Physical and chemical tests were performed over fresh samples (i.e., not subjected to stability tests) of the mixtures MO + FF2 and NE + FF1, to determine how the addition of different concentrations of Fe_3_O_4_ NP affects the physical properties of oils. It must be pointed out that the physical-chemical evaluation of the liquids presented in in this work is not exhaustive and that more work is required to fully understand the behavior of the prepared DNF.

The density and the kinematic viscosity of the prepared DNF were measured finding that the variation of these variables with the increase of the NP concentration was negligible. That lack of change is justified by the very low amounts of NP that were added to the BF. The theoretical variation of the density and viscosity was estimated using the mixture rule and Einstein equation [45], obtaining that for the higher concentration of NP considered in this work (0.35 g/L), the expectable variation of the density would be around 0.01% and the variation of the viscosity would be around 0.1%, what is in agreement with the experimental measurements. Other authors have presented similar results in previous works [12].

Some dielectric properties of the fluids were also evaluated (i.e., Alternate Current (AC) Breakdown Voltage, tangent delta, and resistivity).

**Figure 5:**
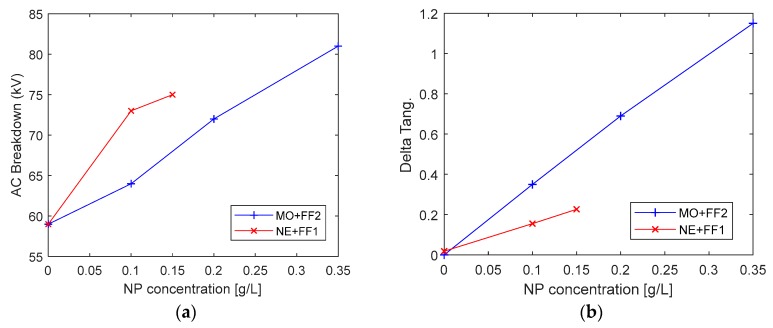
(**a**) AC Breakdown Voltage (50 Hz) and (**b**) Tangent delta of the prepared DNF.

Figure 5a shows the evolution of the breakdown voltage at 50 Hz, measured according to standard IEC 60156 [46]. As can be seen, there is a clear improvement on the dielectric strength of the oil when NP are added to it. This is in agreement with the results shown by many other authors [8,14,15,19,24,47].

The increase of the breakdown voltage, has been generally explained by the EDL theory, according to which when a DNF is subjected to a electric field the NPs act as electron scavengers, attracting the fast electrons in the oil converting them into slower charged particles, what slows down the propagation of the breakdown streamer [21]. Hwang [48] proposes that the effect of dispersing a certain type of NP in a BF on the dielectric breakdown process depends on the NP relaxation time, which is defined as:

$$\tau =\frac{2\varepsilon_{\mathrm{BF}}+\varepsilon_{\mathrm{NP}}}{2\sigma_{\mathrm{BF}}+\sigma_{\mathrm{NP}}}$$

where ε_BF_ and ε_NP_ are the permittivity of the BF and the NP and σ_BF_ and σ_NP_ are their conductivities.

If the relaxation time of the NP is shorter than the time scale of the electrodynamic process being considered, the effect of the NP on the process will be very noticeable, while if the relaxation time is longer than the time scale of the process the NP will have little effect on the breakdown processes. 

Considering the resistivity of the MO and the NE used in this work, and the typical values of the relative permittivity of MO, NE and Fe_3_O_4_ NP reported in the literature (2.2, 3.2 and 7.08·10^−10^), the relaxation times of the prepared DNF have been calculated obtaining times of 7.47·10^−14^ s (for MO) and 7.65·10^−14^ s (for NE). These times are much smaller than the time scale of the breakdown processes, and thus the observed improvement would be justified in the light of Hwang’s theory.

However, some works present experimental results that cannot be justified with the electron-scavenging model and the relaxation time constant [49]. Du et al. [21] propose an alternative explanation for the observed improvement, based on the increase of the shallow trap density of dielectric fluids when NP are present. The authors demonstrate that repeated electron trapping and de-trapping processes could be one of the main charge transport processes in DNF; if the fast electrons, generated in presence of an intense electric field are trapped and de-trapped by the shallow traps when moving from high to low field locations, their speed, and so the speed of the streamer propagation, will drop significantly. The described mechanism explains the enhancement of the dielectric properties observed in DNF based on semi-conductive and insulating NP, and probably plays a role in the results obtained in this work.

Other authors relate the improvement of the breakdown voltage of DNF with the fact that the water molecules present in the oil cluster around the charged NP [3] limiting the hazardous effect of the moisture dissolved in oil; especially if the surfactant that coats the NP is hydrophilic [4]. The samples prepared in this work were subjected to vacuum drying before being tested, so presumably water clustering is not the reason for the observed improvement.

**Figure 6:**
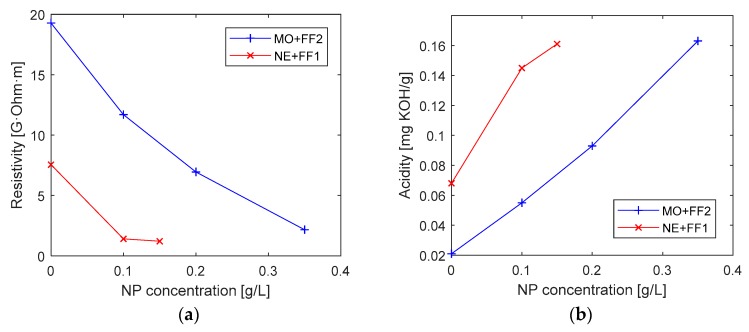
(**a**) Resistivity and (**b**) Acidity of the prepared DNF.

Regarding the tangent delta and resistivity variation, shown in Figure 5b and Figure 6a, it can be seen, that the tangent delta increases with the concentration of NP added to the fluids, while the resistivity decreases. These tendencies, which are equally noticeable in NE and MO-based liquids and are negative for the dielectric performance of the fluid, are probably caused by the presence of conductive NP in the oil. Similar variations for these variables have been shown by other authors [20]. The recommendation of IEEE Std C57.106-2006 is that tangent delta of a service oil should be below 0.5; thus, only the DNF with concentration 0.1 g/L would be within admissible limits.

More research would be required to investigate the change of the tangent delta with the concentration of NP added to the oil; in particular, it would be important to measure the components of the complex permittivity to evaluate the increase of the dielectric loses of the fluid when conductive NP are added. Rajnak [50] evaluated the complex permittivity of a Fe_3_O_4_-based DNF for several concentrations of NP, finding a significant increase of the dielectric losses as higher concentrations of conductive NP are added to the oil. The author demonstrates that the observed increase is mainly due to the polarization and relaxation processes that take place on the NP-counterions system when the DNF is subjected to an electric field, and in much lower extent to the increase of the electric conduction in the fluid.

The fluid’s relative permittivity also influences the tangent delta. Miao et al. [51] investigated the permittivity variation of DNF, finding that the permittivity of DNF rises slightly with the NP concentration; the authors developed a physical model to explain the permittivity change, based on the analysis of the NP inner polarization in presence of an electric field.

Finally, the evolution of the acidity of the oils with the increase of the NP concentrations was measured. As shown in Figure 6b as higher concentrations of NP are dispersed in the BF, the acidity of the DNF is higher. This is not a positive factor, because the presence of acids in oil accelerates its ageing rate and can lead to formation of sludges, which are harmful for the solid insulation. However, it should be noted that the measured values are still far from the limit value recommended by IEEE Std C57.106-2006 [44] for a service MO, i.e., 0.2 mg KOH/g, and also from the level of 0.4 mg KOH/g where sludges may start to form. The increase of the acidity might be caused by the presence of the surfactants in the NP dispersions used to prepare the DNF, although more research would be necessary to understand the causes of that increase.

### 3.2. Results for the Stability Tests

#### 3.2.1. Visual Inspection

During the experiments, a visual inspection of the samples was carried out daily to corroborate that aggregates had not formed in the dispersion. The formation of precipitates was considered as an indication of stability loss. Table 3 summarizes the results of the visual inspection analysis. The column “Stability” indicates the time that elapsed without the sample showing any observable precipitate or aggregate.

As explained in Section 2.3 the testing period for the samples which were subjected to temperatures 50–110 °C was two months. After this time if the samples had not precipitated, they were taken out from the oven and the test stopped. The sample that remained at ambient temperature was tested for 10 months.

**Figure 7:**
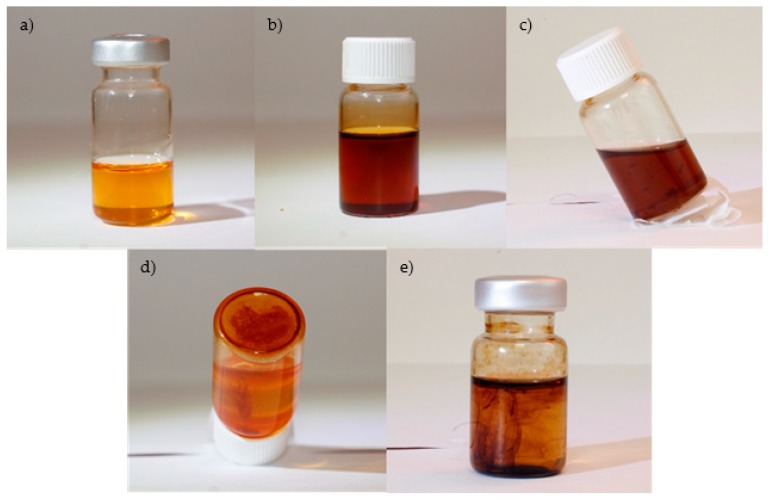
Appearance of the solutions after the tests (From top left to bottom right): (**a**) Type 1, NE + FF1 (0.1 g/L), (**b**) Type 1, MO + FF2 (0.1 g/L) (**c**) Type 2, precipitate suspended, (**d**) Type 3, precipitate at the bottom of the vial, (**e**) Type 4, NP could not be dispersed.

The appearance of the solutions at the end of the stability tests was analyzed, distinguishing between five different final appearances:

- **Type 1**: Samples that remain with a good dispersion after the testing period. Examples of this can be seen in Figure 7a, which corresponds to a dispersion of NE with NP at concentration 0.1 g/L. and Figure 7b, which shows a sample of MO with NP at concentration of 0.1 g/L after 2 months of testing.
- **Type 2**: Samples in which, after some time, the nanoparticles form a precipitate that remains suspended in the solution. An example of this can be seen in Figure 7c.
- **Type 3**: Samples in in which the nanoparticles form a precipitate at the bottom of the vial. The precipitate is solid and is deposited and adhered at the bottom of the vial. An example of this can be seen in Figure 7d.
- **Type 4**: Samples in which the nanoparticles could not be dispersed by ultrasounds during the manufacturing stage. An example of this can be seen in Figure 7e.

**Figure 8:**
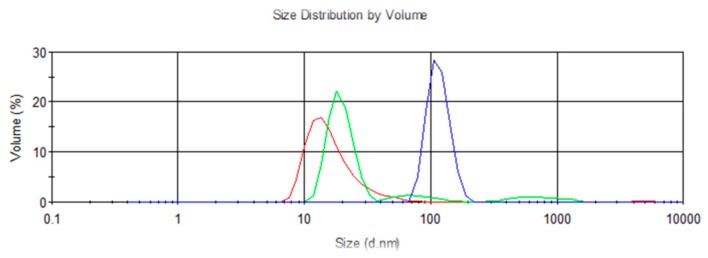
Measured particle-size distribution by volume: Red: MO + FF2, Green: NE + FF2, Blue: NE + FF1. Testing temperature 25 °C.

According to the results obtained with the visual inspection, the following conclusions can be extracted:

- The mixture of the NE and the NP dispersion FF1 (0.1 g/L) was the liquid that presented the best long-term stability, showing a good performance at all tested temperatures. Even the sample that was subjected to 110 °C for two months remained stable showing no visible aggregates or deposits at the end of the test. The same solution remained stable at ambient temperature for at least 10 months (after 10 months the test was discontinued), which is a long time compared with the ones reported in the literature.
  On the other hand, the mixture of the NE with the NP dispersion FF2 led to a less stable fluid. At ambient temperature visible NP aggregates appeared after 3 months of testing. The samples that were subjected to higher temperatures also showed visible NP aggregates after 7 weeks of testing, in the case of the sample tested at 50 °C, and solid deposits after 4 weeks and 2 days when tested at 60 and 80 °C.
- In the case of MO-based samples, the mixture of MO and the NP dispersion FF2 (0.1 g/L) led to a fluid which was highly stable at ambient temperature. After 10 months of testing the sample did not show any visible aggregates or deposits. The stability of this mixture was also good when subjected to moderate temperatures (50 and 60 °C), for 2 months. However, visible NP aggregates appeared in the fluid after 13 days when the sample was tested at 80 °C.
  The mixture of MO and the dispersion FF1 did not lead to a homogeneous fluid after the ultrasounds stirring and solutions similar to the one shown in Figure 8e were formed instead.

As explained before, the magnetic nanoparticles need to be stabilized in the carrier liquid, because they tend to form aggregates due to Van der Waals forces, as a solution to reduce their high surface energy. The suppliers of the NP dispersions solve this problem by adding a dispersing agent which usually is a surfactant (for example a fatty acid). When the nanofluid is diluted in the oil the objective is to maintain the dispersion stable. There is a kinetic stabilization against gravitational forces, related with Brownian motion or the viscosity of the medium, and aggregation stabilization through the formation of an adsorption layer. When diluted in the oil, the stability of the dispersion depends on how the aggregation stabilization operates in the system [52,53].

According to the experimental observations it is clear that the temperature is a variable that has a high impact on the stability of the analyzed DNF. The worsening of stability in the DNF when increasing the temperature may be related with the changes that occurs in the Brownian Movement of the NP in the DNF. When the temperature increases, the speed of the NPs increases as well, what produces a greater number of collisions and these occur with greater energy, as [54] shows. Another effect that might explain the impact of the temperature on the DNF stability is the fact that at higher temperatures the surfactant might suffer some degradation losing its properties and allowing the agglomeration of the NP, as indicated in [38]. Finally, the viscosity of the fluid decreases what favors the formation of aggregates.

Additionally, the results demonstrate that not all the materials combine equally well with the different BFs. In this sense, the NE, which is a fluid with a certain degree of polarity, seem to form more stable dispersions when a polar surfactant, as the carboxylic acid present in FF1, is added to the mixture. Conversely the surfactant present in FF2 seem to be more suitable to produce MO-based DNF.

As explained before, vials in which samples of Kraft paper coexisted with the DNF were prepared and subjected to the same experimental conditions than the samples listed in Table 3. The results of these tests are not shown in Table 3, since there were no significant differences (i.e., above 24 h) between the stability times of these samples and those prepared without paper. This appreciation has been confirmed by particle-size measurement which are shown just below in the next section.

#### 3.2.2. Particle-size Measurements

Particle-size measurements were carried out on all the samples that showed certain stability at the end of the testing period (ten months). This includes the fluids obtained from the combinations NE + FF1, NE + FF2 and MO + FF2. The fluid MO + FF1 was not included in the particle-size study since, as was explained before, that combination did not lead to homogeneous dispersions.

**Figure 9:**
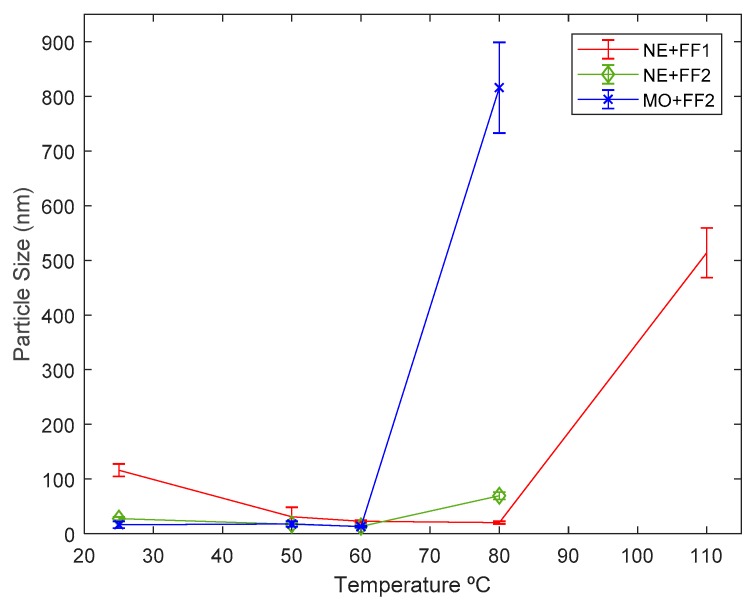
Average particle size of the DNF at the end of the testing period and error bar (i.e., 2 months for T > 30 °C and 10 months for T = 25 °C). FWHM is presented as the error bar.

Figure 8 shows, as an example, three of the measuring registers. It can be observed that the measures show a distribution of particle sizes centered around a central point. Table 4 shows the main peak observed in the volume distribution registered for each of the analyzed samples, as well as the full width at half maximum of the size distribution (FWHM). According to the equipment, the main peak is responsible for more than 98% of the sample scattering. The results have been plotted in Figure 9 as well.

From the obtained results, it can be deduced that in general the formation of aggregates increases with temperature or time, this effect being observed mainly at the highest temperatures tested. As the temperature increases, the surfactant layer increases its capacity for elastic deformation during collisions between particles, reducing its hydrodynamic radius and increasing the mean available volume [55]. This may favor aggregation by allowing a close interaction between particles through Van der Waals interactions.

As can be seen, for the case of dispersion MO + FF2, all the particle-size measurements are around the 10 nm specified by the supplier, which in practice means that no aggregation has occurred. From 80 °C it could be said that the particle size shoots up, making the mixture unstable, what agrees with the conclusions of the visual inspection. It should be noted that we have also measured the size distribution of the ferrofluids. Mean peak values of around 22 nm (FWHM of 9 nm) for FF1 and 18 nm (FWHM of 10 nm) for FF2 were obtained.

In the case of the measures performed on NE + FF1 samples subjected to stability tests, an increase on the particle size can be observed as the testing temperature rises. Although the visual inspection seemed to indicate that all the samples of the liquid remained stable for the 2 months period, some of the particle-size measurements showed appreciable changes. In particular, for the samples that were kept at 25 °C for ten months, the particle-size measurement showed that NP aggregates ten times larger than the original particle size were present in the samples of the fluid, what implies that some degree of aggregation. As can be seen the samples remained stable when subjected to temperatures within 50 and 80 °C although significant increases in the particle sizes can be observed on the samples that had been subjected to stability tests at 110 °C.

Finally, for the dispersion of NE + FF2, it can be observed that although the nanoparticles kept a size consistent with the information of the supplier, visible deposits were present in the samples at the end of the tests. This can be due to the fact that, while there are NP that remain dispersed in the oil, others form aggregates in the system. This would result in a lower concentration of particles in the DNF compared to the calculated ones.

Several DNF samples with incorporated Kraft paper were analyzed also by DLS to check if there was any influence over size distribution. Results are not shown as the obtained values are of the same magnitude order than the observed for DNF samples. For example, NE + FF1 with Kraft paper kept at 25 °C showed the size distribution main peak at 156 nm (FWHM of 34 nm), but NE + FF2 or MO + FF2 with Kraft paper, also kept at 25 °C, showed their main peaks at only 19 nm (FWHM of 7 nm) and 13 nm (FWHM of 5 nm) respectively, in accordance with DNF measurements without Kraft paper.

The only difference between samples with paper and without paper is that in the ones without paper the precipitate was deposited at the bottom of the vial, while in the samples with paper the precipitate was deposited on the surface of the paper. This observation seems to indicate that the presence of paper has not a big impact on the long-term stability of DNF. This is be a positive aspect that can facilitate the application of these fluids to service transformers.

According to these results, it seems clear that the long-term stability of DNF depends strongly on the working temperature and that all the analyzed mixtures become less stable when they are subjected to higher temperatures for long periods of time. Although this aspect can be a major problem for the application of DNF to service transformers it had not been pointed out by any other author before [30,56].

## 4. Conclusions

In this paper, the stability of several DNF for use in transformers was evaluated. According to the experimental evidence, it can be concluded that although the application of ultrasonic stirring leads to stable DNF with superior dielectric properties compared with the corresponding BF, some of these liquids lose stability when subjected to high temperatures for long periods of time. The loss of stability could be related with the loss of the bound between the surfactant and the NP or to some other factors.

Although most published works on transformer DNF evaluate the stability of the liquids at ambient temperature, the service temperature is a key factor that must be considered in the stability tests, since fluids that perform well at ambient temperature become non-stable above certain temperature ranges. The stability of newly developed DNF must be tested at typical transformer working conditions to guarantee that they can withstand the operating temperatures they will be subjected to. Other aspects, such as the presence of cellulosic insulation do not seem to affect the stability of the samples significantly.

The materials used to produce the DNF also have a major influence on the stability; in this sense, carboxyl functionalized surfactants seem to be more adequate for NE oils, whereas non-carboxylic surfactant of FF2 dissolve better the DNF in low polarity fluids, as MO. Regarding the methodologies that can be applied to the evaluation of the stability of DNF, it has been shown that visual inspection and particle-size measurement can be used as complementary methods to get information about the formation of precipitates in the dispersions and the aggregation phenomena. Although visual inspection has been generally used to analyze the stability of DNF by many authors, that method might fail in detecting moderate aggregation of NP within the fluids. On the other hand, Zeta potential is not a suitable method to characterize the stability of these types of liquids as, given their non-polar nature of the BF and the NP concentrations typically used for this application, the NPs do not attach a sufficient amount of charge to their surfaces to enable the measure.

As a general conclusion it can be said that, although the application of DNF to power transformers is promising and the development of these materials will probably lead to more compact and reliable machines in the future, more work is still needed to improve the long-term stability of these liquids under transformer typical operating temperatures.

## Figures and Tables

**Table 1:** Main properties of the BF included in the study. Taken from [39,40].

Property	NE Bioelectra	MO Nytro 4000X
**Physics and Chemicals**		
Kinematic Viscosity (40 °C)	39.2 cSt	9.1 cSt
Flash Point	334 °C	146 °C
Pour point	−21 °C	−54 °C
Density (20 °C)	0.91 g/cm^3^	0.866 g/cm^3^
Appearance	Clear and bright	Clear, free from sediment
Water Content	100 mg/kg	<20 mg/kg
**Electricals**		
Breakdown voltage	65 kV	>70 kV
Dissipation Factor	0.03	<0.001
**Oxidation Stability**		
Total acidity	0.2 mg KOH/g	<0.01 mg KOH/g
Sludge	0.01%	<0.01%
Dissipation Factor at 90 °C, 50 Hz	0.05	<0.01

**Table 2:** Physical properties of FF1 and FF2.

Physical Property	FF1	FF2
Nanoparticle	Fe_3_O_4_	Fe_3_O_4_
Nanoparticle size	≈10 nm	≈10 nm
Carrier Liquid	Hydrocarbons	Hydrocarbons
Surfactant	Carboxilic acid	-
Solid Content	60%	50%
Density (20 °C)	1.21 g/cm^3^	1.04 g/cm^3^
Dynamic Viscosity (27 °C)	87 cP	80 cP
Manufacturer	Magnacol	MAGRON

**Table 3:** Results of the stability tests according to the visual inspection.

Oil	NP (0.1 g/L)	Test Temperature	Stability	Final State
Bioelectra (NE)	FF1	25 °C	10 months	Type 1
		50 °C	2 months	Type 1
		60 °C	2 months	Type 1
		80 °C	2 months	Type 1
		110 °C	2 months	Type 1
	FF2	25 °C	3 months	Type 2
		50 °C	7 weeks	Type 2
		60 °C	4 weeks	Type 3
		80 °C	2 days	Type 3
Nynas Nitro 4000X (MO)	FF1	25 °C	Unstable	Type 4
		50 °C	Unstable	Type 4
		60 °C	Unstable	Type 4
		80 °C	Unstable	Type 4
	FF2	25 °C	10 months	Type 1
		50 °C	2 months	Type 1
		60 °C	2 months	Type 1
		80 °C	13 days	Type 3

**Table 4:** Stability tests according to particle size measurements. FWHM is the full peak width at half maximum.

Oil	NP (0.1 g/L)	Test Temperature	Stability	Main Peak (nm)	Peak FWHM (nm)
Bioelectra (NE)	FF1	25 °C	10 months	116	23
		50 °C	2 months	31	33
		60 °C	2 months	23	4,6
		80 °C	2 months	20	4,8
		110 °C	2 months	514	91
	FF2	25 °C	3 months	28	6,2
		50 °C	7 weeks	17	8,3
		60 °C	4 weeks	13	6,2
		80 °C	2 days	69	13.4
Nynas Nitro 4000X (MO)	FF2	25 °C	10 months	16	12,3
		50 °C	2 months	18	10.5
		60 °C	2 months	13	2,3
		80 °C	13 days	816	166
